# Nicotine exposure during adolescence alters the rules for prefrontal cortical synaptic plasticity during adulthood

**DOI:** 10.3389/fnsyn.2012.00003

**Published:** 2012-08-02

**Authors:** Natalia A. Goriounova, Huibert D. Mansvelder

**Affiliations:** Department of Integrative Neurophysiology, CNCR, Neuroscience Campus Amsterdam, VU UniversityAmsterdam, Netherlands

**Keywords:** adolescence, nicotine, prefrontal cortex, STDP, mGluR, nAChR, cognitive behavior

## Abstract

The majority of adolescents report to have smoked a cigarette at least once. Adolescence is a critical period of brain development during which maturation of areas involved in cognitive functioning, such as the medial prefrontal cortex (mPFC), is still ongoing. Tobacco smoking during this age may compromise the normal course of prefrontal development and lead to cognitive impairments in later life. In addition, adolescent smokers suffer from attention deficits, which progress with the years of smoking. Recent studies in rodents reveal the molecular changes induced by adolescent nicotine exposure that alter the functioning of synapses in the PFC and underlie the lasting effects on cognitive function. In particular, the expression and function of metabotropic glutamate receptors (mGluRs) are changed and this has an impact on short- and long-term plasticity of glutamatergic synapses in the PFC and ultimately on the attention performance. Here, we review and discuss these recent findings.

Adolescence is an important developmental period when a child has to make a transition to an independent status of adult. Such transition demands an ability to take risks and a taste for novelty but also emergence of self-control and more adult decision-making strategies. Also, brain development is not complete by adolescence, especially maturation of areas involved in cognitive functioning, such as the medial prefrontal cortex (mPFC), is still ongoing. Brain development continues throughout adolescence, though the speed and timing of maturation varies for different brain areas (Gogtay et al., 2004). Subcortical limbic structures important for emotional processing, such as hypothalamus, midbrain dopamine areas, nucleus accumbens, dorsal, and ventral striatum and amygdala, experience a major developmental boost around the onset of puberty (Sowell et al., 2003; Casey et al., 2005). Their maturation is important for social and sexual behaviors and is triggered by pubertal hormones. In contrast, development of frontal cortical areas of the brain, responsible for cognitive control over behavior, depends on age and experience and continues throughout adolescence and into adulthood (Sowell et al., 2003; Giedd, 2004). Thus, during adolescence emotional drive has already become very strong while cognitive self-control and adult decision-making strategies still are developing. Thereby, brain development may be responsible for characteristic adolescent traits—uncontrollable mood swings, impulsivity, risk-taking, and peer-directed social interactions (Orr and Ingersoll, 1995; Spear, 2000; Galvan et al., 2007). Although indispensable for transition from child to independent status of adult, these traits hold hazards. Indeed, risk-taking behavior, so typical for adolescents, is associated with high rates of mortality and morbidity among young people (Grunbaum et al., 2004).

The impulsive, peer-influenced nature of adolescent choices sets the stage for experimenting with drugs of abuse. Since nicotine is one of the most socially accepted drugs in our society, the first choice usually falls on tobacco smoking. According to a recent study conducted in 41 countries in Europe and North America, 19% of 15-year olds smoke at least once a week and 30% report experimenting with cigarettes before the age of 14 (Currie et al., 1993). Serious health risks of smoking are well-known: smoking leads to millions of premature deaths worldwide and tobacco smoking has been marked as an epidemic disease (Peto et al., 1999). Nicotine is also a psychoactive and addictive substance that directly acts on brain areas involved in emotional and cognitive processing. Early exposure to nicotine during adolescence may disturb the normal course of brain maturation and have lasting consequences for cognitive ability, mental health, and even personality (Brown et al., 1996; Choi et al., 1997; Richards et al., 2003; Brook et al., 2004; Deas, 2006). In humans, the PFC shows delayed development with respect to other cortical areas during adolescence with delayed thinning of cortical grey matter, most likely reflecting fine-tuning of synaptic contacts (Gogtay et al., 2004; Sowell et al., 2004; Casey et al., 2005). Rearrangement of local inhibitory inputs and decreases in synaptic densities and branch points of excitatory connections between pyramidal neurons occur within the developing PFC (Woo et al., 1997; Cruz et al., 2003). Spike-timing-dependent modifications are likely to be important for cortical development, map plasticity, and functioning of neural networks: correlated inputs lead to strengthening of connections (LTP) while uncorrelated inputs lead to weakening (LTD) and pruning of unused synapses (Bi and Poo, 2001; Song and Abbott, 2001; Feldman and Brecht, 2005). Here, we highlight recent findings that start to uncover causal relations between nicotine exposure during adolescence and cognitive deficits in later life, with an emphasis on synaptic adaptations and altered rules for synaptic plasticity in prefrontal networks.

## Immediate effects of nicotine exposure

Nicotine activates nicotinic acetylcholine receptors (nAChR), which take part in cholinergic signalling. Twelve genes have been identified encoding neuronal nicotinic receptors (for review see Le Novere et al., 2002; Millar and Gotti, 2009). In the central nervous system 9 α-subunits (α2–α10) and 3 β-type subunits (β2–β4) are expressed. These subunits assemble in different stoichiometries to form pentameric channels, and subunit compositions of nAChRs vary depending on the brain region (for review see Grady et al., 2002; Le Novere et al., 2002; McGehee, 2002; Alkondon and Albuquerque, 2004; Wonnacott et al., 2005; Mineur and Picciotto, 2008; Millar and Gotti, 2009). Nicotinic AChRs are cation selective channels that permit the flow of Na^+^, K^+^, and Ca^2+^ across the membrane, which leads to depolarizing currents and activate neurons (McGehee and Role, 1995; Millar and Gotti, 2009).

Modulation of PFC activity by nAChRs will depend on which cell types express nAChRs and what subunits they are made of. In the PFC, nAChR expression is found across all layers (Gioanni et al., 1999; Poorthuis et al., 2012). Activation of nAChRs can alter pyramidal neuron activity by two mechanisms: presynaptically enhancing glutamatergic inputs or by activating postsynaptic receptors directly (Poorthuis et al., 2012). Recently we measured nAChR activation by making whole-cell recordings from PFC pyramidal neurons in the different layers and used wild-type, β2-null, or α7-null mice as well as pharmacological tools to determine the nAChR subunits involved. We showed that PFC pyramidal neurons across cortical layers show a differential pattern of postsynaptic nAChR activation: layer II/III pyramidal neurons do not contain nAChRs, layer V pyramidal neurons contain α7 nAChRs, and layer VI pyramidal neurons are modulated by β2^*^ nicotinic receptors (Poorthuis et al., 2012). Also presynaptic glutamatergic inputs can be modulated by nicotine (Gioanni et al., 1999; Lambe et al., 2003; Couey et al., 2007; Poorthuis et al., 2012). We found that this presynaptic regulation is specific to layer V, only moderately present in layer VI and not present in layer II-III (Poorthuis et al., 2012).

In addition to direct activation of PFC pyramidal neurons by nAChRs, PFC GABAergic interneurons across all layers are also directly activated by nAChR stimulation (Poorthuis et al., 2012). Increased inhibition through activation of nAChRs expressed by interneurons has been found in many different brain regions (Jones and Yakel, 1997; Xiang et al., 1998; McQuiston and Madison, 1999; Alkondon et al., 2000; Ji and Dani, 2000; Mansvelder et al., 2002; Gulledge et al., 2007). Interneurons form a highly diverse group of cells with distinct roles in cortical computation (Kawaguchi, 1993; Markram et al., 2004). Fast-spiking cells target the perisomatic region of pyramidal neurons (Kawaguchi and Kubota, 1997; Kawaguchi and Kondo, 2002) and are therefore thought to be involved in regulating the activity window of pyramidal neurons. Feedforward inhibition in the PFC plays an important role in the integration of hippocampal inputs, which enter the PFC through superficial layers (Jay and Witter, 1991; Tierney et al., 2004). Fast-spiking cells in PFC layer II-III contain α7 nAChRs, as do as about half of the fast-spiking cells in layer V (Poorthuis et al., 2012). nAChR activation on fast-spiking interneurons in PFC layer II/III may alter processing of hippocampal inputs.

Somatostatin-positive cells target distal dendritic regions (Kawaguchi and Kondo, 2002; Silberberg and Markram, 2007) and can mediate disynaptic inhibition between pyramidal neurons (Silberberg and Markram, 2007; Kapfer et al., 2007). Regular-spiking and somatostatin-positive cells in PFC layer II-III and V are positive for nAChRs, suggesting that nAChRs play an important role in modulating feedback inhibition among pyramidal neurons in these layers (Poorthuis et al., 2012).

Finally, we showed that the network activity in the PFC in response to bath application of ACh is dominated by β2 nAChRs activation. Receptors of this subtype are expressed by pyramidal neurons in layer VI, glutamatergic inputs to layer V and VI and by interneurons in all layers of the PFC. In summary, β2 containing nAChRs stimulate both excitatory and inhibitory neurons in deep layers, while in layer II/III only interneurons are activated (Poorthuis et al., 2012). Thus, the net result of nicotinic receptor stimulation is the increased inhibitory transmission in superficial PFC layers, whereas in deep layers it also leads to activation of pyramidal neurons. This pattern of activation alters the information processing in prefrontal networks and can directly alter rules for plasticity (Couey et al., 2007).

One of the forms of long-term plasticity, spike-timing dependent plasticity (STDP), is based on Hebb's postulate often summarized as “*Cells that fire together, wire together*” (Hebb, 1949). It attempts to explain associative learning, in which nearly simultaneous activation of cells increases the strength of synaptic contacts between those cells. This type of plasticity is thought to play an important role in cortical development during critical stages and underlie some forms of learning (Feldman et al., 1999; Feldman and Brecht, 2005; Letzkus et al., 2007). In PFC, STDP can be directly modulated by nicotine (Couey et al., 2007; Goriounova and Mansvelder, 2012). We showed that in adolescent rats, STDP in layer V pyramidal neurons is strongly reduced if nicotine is applied to PFC slices during induction protocol (Goriounova and Mansvelder, 2012). In PFC layer V, the increase in inhibition dominates the effects of nicotine on synaptic plasticity (Couey et al., 2007). Nicotine-stimulated inhibition reduces dendritic calcium signaling and renders postsynaptic activity in layer V pyramidal cells insufficient to induce potentiation (Couey et al., 2007). In layer II/III, the layer where the synaptic inputs were stimulated to induce STDP, nAChR activation results predominantly in activation of interneurons (Poorthuis et al., 2012). Thus, nicotine-induced increase in inhibitory transmission can explain the decrease in spike-timing dependent potentiation in adolescent rats.

## Upregulation of nAChR and synaptic mGluR expression

Adolescents may be more vulnerable to nicotine addiction due to greater positive effects nicotine has on adolescents than adults, whereas the negative effects associated with nicotine, such as withdrawal are smaller in adolescents (O'Dell, 2009). Nicotine administration during, but not following adolescence, has long-lasting effects on cognitive, addictive, and emotional behavior in rats (Adriani et al., 2003; Iniguez et al., 2008; Counotte et al., 2009, 2011). Furthermore, adolescent animals are more sensitive to nicotine conditioned place preference than adults and show this at lower nicotine doses (Vastola et al., 2002; Belluzzi et al., 2004; Shram et al., 2006; Brielmaier et al., 2007; Kota et al., 2009). Adolescent nicotine exposure leads to acute and longer-lasting changes in nAChR binding (Abreu-Villaca et al., 2003; Doura et al., 2008) and function (Kota et al., 2009) in brain regions such as cortex and striatum. We recently found that the adolescent rodent brain is more sensitive to nicotinic receptor upregulation in the medial PFC (mPFC) than adults (Counotte et al., 2012). Naïve rats show an age-related decrease in ^3^H-epibatidine labeled high-affinity nicotinic receptors in the mPFC, but not in occipital cortex. Adolescent, but not adult nicotine exposure increases ^3^H-Epi-binding of mPFC receptors on the first day of abstinence following 10 days of nicotine injections. This is paralleled by an mPFC-specific increase in expression of nAChRs containing α4 and β2 (but not α5) subunits or high-affinity nAChRs. The increased expression of high-affinity nAChRs in adolescents is accompanied by an increase in nicotine-stimulated GABAergic synaptic transmission in the mPFC (Counotte et al., 2012).

One of the first and most common cellular adaptations following chronic nicotine exposure is the upregulation of nicotinic receptor levels (Dani and Bertrand, 2007). Especially α4β2 type of nAChRs appears to be selectively upregulated via posttranslational mechanisms (Miwa et al., 2011). The upregulation of α4β2 nAChRs by chronic nicotine treatment has been replicated many times in numerous systems—transfected cell lines, neurons in culture, brain slices, and smokers' brains (Wonnacott, 1990; Fu et al., 2009; Lester et al., 2009; Marks et al., 2011; Miwa et al., 2011). Upregulation is not accompanied by an increase in nAChR subunit mRNA (Marks et al., 1992), instead it leads to increased nAChR protein levels resulting from posttranslational mechanisms (Govind et al., 2009; Marks et al., 2011). According to one view on nAChR upregultation, nicotine acts intracellularly as a selective pharmacological chaperone of acetylcholine receptor (Lester et al., 2009). It stabilizes nAChRs during assembly and maturation and this stabilization is most pronounced for the high-affinity nAChR containing α4β2 subunits. Other possible mechanisms underlying nAChR upregulation can result from increased cell surface turnover, increased receptor trafficking to the surface, changes in subunit stoichiometry or nAChR conformational changes (Govind et al., 2009). All these proposed mechanisms have two features in common: they are posttranslational and involve upregulation of high-affinity nAChR containing α4β2 subunits. Indeed, we found that specifically high-affinity nicotinic receptors containing the α4 and β2 subunits were upregulated in the adolescent PFC shortly following nicotine exposure. This upregulation was paralleled by a functional elevation in nicotine-stimulated GABAergic transmission, indicating that functional surface nAChRs are upregulated as well (Counotte et al., 2012).

Given that pyramidal neurons and excitatory projections in layer II/III of the PFC do not express nAChRs (Poorthuis et al., 2012), the functional consequence of α4β2 nAChR upregulation on interneurons in layer II/III will be an increased inhibitory transmission in superficial PFC layers. In the deep layers of the PFC, β2 subunits are expressed by both interneurons as well as layer VI pyramidal neurons and excitatory inputs to layer V pyramidal neurons. An upregulation of these receptors will lead to a combined increase in activation of pyramidal neurons and interneurons. Since β2-containing nAChRs in the mPFC control attention performance (Guillem et al., 2011) this may have functional implications for maturation and function of the prefrontal network. During chronic nicotine exposure of the adolescent PFC, the pattern of activity in prefrontal network may gradually shift toward activation of excitatory neurons in deep layers in the context of increased overall inhibition. This may affect plasticity and refinement of cortical connections (Couey et al., 2007), even though GABAergic transmission by itself was not affected directly following nicotine treatment during adolescence (Counotte et al., 2012). Still, we find that also directly following nicotine exposure during adolescence *in vivo*, STDP of glutamatergic synapse strength is blocked (Goriounova and Mansvelder, 2012). Since in these experiments assessing STDP immediately following nicotine treatment during adolescence nicotine was not applied, this can not explain the reduced STDP we observed.

**Figure 1:**
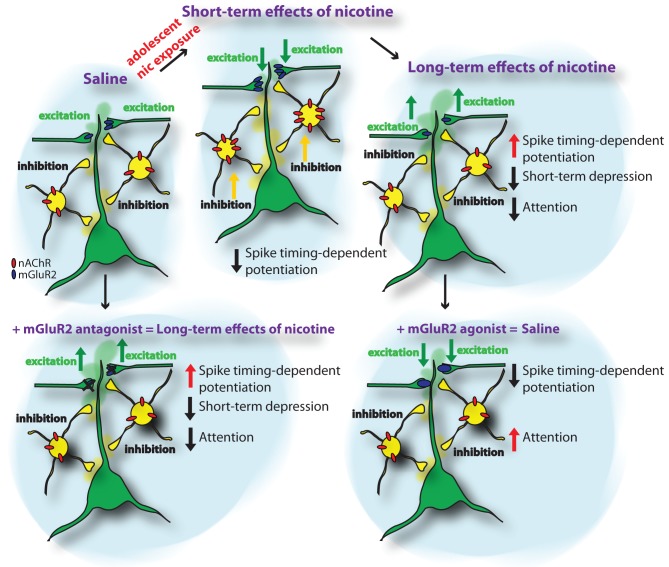
**Schematic representation of the short-term and long-term adaptations in PFC neuronal networks caused by nicotine exposure during adolescence.** The upper panels show the sequence of adaptations in nAChR and mGluR2 protein levels and the resulting changes in inhibition and excitation and attention behavior from control conditions (saline) to nicotine exposure during adolescence (short-term effects of nicotine) and 5 weeks following nicotine exposure (long-term effects of nicotine). The lower panels show the effects of mGluR2 agonists and antagonists in saline and nicotine-exposed animals. Applying mGluR2 antagonists to the adult mPFC reduces depression of glutamatergic synapses and reduces attention performance of the animal. Providing mGluR2 agonists to the mPFC of adult rats that were exposed to nicotine during adolescence normalizes synaptic depression and spike-timing-dependent potentiation of glutamatergic synapses and improves attention performance.

How is spike-timing-dependent potentiation affected by nicotine exposure during adolescence? In addition to an upregulation of nAChRs, we recently found in a large-scale iTRAQ-based proteomics screen of synaptic protein levels in the PFC that metabotropic glutamatergic receptors type 2 (mGluR2) are significantly upregulated during adolescent nicotine exposure (Figure 1) (Counotte et al., 2011). This short-term upregulation is followed by a long-term decrease in mGluR2 levels 5 weeks after the nicotine exposure during adolescence. As will be discussed later, this pattern of mGluR2 expression leads to opposing effects on glutamatergic transmission and plasticity in PFC.

Metabotropic GluR2s are located presynaptically on glutamatergic synapses and their activation reduces the probability of glutamate release. Thereby, an upregulation of mGluR2 levels diminishes activity of excitatory glutamatergic synapses in the PFC. Thus, increases in functional nAChR on inhibitory neurons and increased nicotine-stimulated excitation in deep layers of the PFC may be counteracted by reduced excitatory synaptic activity mediated by increased mGluR2 activity. Blocking mGluR2s with MPPG restored spike-timing-dependent potentiation following nicotine exposure during adolescence back to levels observed in animals that received saline treatment during adolescence (Goriounova and Mansvelder, 2012). This suggests that the upregulation of presynaptic mGluR2s after nicotine exposure during adolescence alters the rules for STDP in PFC networks.

In summary, the net result of nicotine in the short-term in adolescent mPFC that is chronically exposed to nicotine, very likely amounts to persistently enhanced levels of inhibition across all cortical layers possibly combined with some increase in thalamocortical glutamate release by presynaptic (or preterminal) nAChRs in deep layers. The latter effect, however, is most likely counteracted by elevated presynaptic mGluR2 levels. This general increase in inhibition plays a role in the plasticity and refinement of cortical connections (reduced STDP in nicotine) and thus may have functional implications for cognitive processing and maturation of prefrontal network.

## Long-term consequences

Smoking during adolescence is associated with disturbances in working memory and attention as well as reduced PFC activation (Jacobsen et al., 2005, 2007; Musso et al., 2007). Smoking is also a prospective risk factor for impaired cognitive function in later life; heavy smoking predicts incident cognitive impairment and decline (Cervilla et al., 2000; Kalmijn et al., 2002; Richards et al., 2003). In animal studies exposure during adolescence induces stronger changes in gene expression in the PFC than during other periods of development and adulthood (Schochet et al., 2005, 2008; Polesskaya et al., 2007). The adolescent PFC shows nicotine response in gene regulation involved in vesicle release, signal transduction, cytoskeleton dynamics, and transcription, suggesting the role of chronic nicotine exposure in initiating long-term structural and functional adaptations (Polesskaya et al., 2007). The expression of key molecules involved in plasticity is also altered in the PFC by adolescent nicotine exposure. Acute nicotine induces increases in the expression of the dendritically targeted dendrin mRNA in PFC of adolescent but not adult animals. Dendrin is an important component of cytoskeletal modifications at the synapse and therefore can lead to unique plasticity changes in the adolescent PFC (Schochet et al., 2008). Lasting synaptic adaptations involve activation of intracellular signaling pathways and enzymes such as extracellular regulated protein kinase (ERK) and cAMP response element binding protein (CREB). Specifically in the PFC, increases in phosphorylation of both these enzymes were found after repeated nicotine exposure (Brunzell et al., 2003). Also changes in macromolecular constituents indicative of cell loss (reduced DNA) and altered cell size (protein/DNA ratio) can be seen in cortical regions of rodents after adolescent nicotine treatment (Trauth et al., 2000). Further, repeated nicotine exposure also alters the structure of neurons in mPFC: it increases both dendritic length and spine density (Brown and Kolb, 2001). Long-term changes have been observed in dendritic morphology of specific subpopulations of pyramidal neurons and these structural changes depended on the age of drug exposure (Bergstrom et al., 2008).

Also on the behavioral level, nicotine during adolescence leads to persisting deficits. Adolescent, but not adult, nicotine treatment reduces accuracy of correct stimulus detection in a visuospatial attentional task, with an increase in premature and time-out responding that suggests impaired attention and lack of impulsive control which is part of normal adolescent maturation (Counotte et al., 2009). Similar nicotine-induced deficits have been found in a serial pattern learning paradigm (Fountain et al., 2008). In a recent study, chronic nicotine exposure during adolescence produced long-lasting impairments in contextual learning that were observed during adulthood, whereas adult chronic nicotine exposure did not (Portugal et al., 2012).

Taken together, these studies in rodents show that nicotine exposure during adolescence induces significant changes in gene expression, neuronal morphology, and behavior in PFC. Thus, nicotine does not only change cholinergic signalling by altering nicotinic receptor levels in the adolescent PFC, but can also lead to secondary adaptations involving structural and functional changes in cognition. What are the changes that underlie the changes in cognitive performance?

## Lasting synaptic adaptations in the PFC

In adult rodents that were exposed to nicotine during adolescence only a handful of proteins show long-term adaptations following adolescent nicotine exposure that persisted into later life. Nicotinic AChR levels in the PFC returned to baseline 5 weeks following adolescent nicotine exposure (Counotte et al., 2012). In contrast, mGluR2 levels show a strong down-regulation at this time (Counotte et al., 2011). Reduced mGluR2 function in mPFC synapses resulted in impaired attention performance. Stimulating mGluR2s with specific agonists improved attention performance in animals that were exposed to nicotine during adolescence (Counotte et al., 2011). Interestingly, the association between changes in mGluR2 signalling and nicotine exposure is not limited to the PFC. Also in other brain areas involved in reward processing such as ventral tegmental area (VTA) and the nucleus accumbens (NAcc) lasting adaptations in mGluR2 function follow nicotine exposure and were found to affect rewarding properties of nicotine (Helton et al., 1997; Kenny et al., 2003; Kenny and Markou, 2004; Liechti et al., 2007). In these brain areas, activation of mGlu2/3 receptors decreases nicotine self-administration (Liechti et al., 2007), and they play an important role in the development of drug dependence and the expression of the negative affective state observed during withdrawal (Pilc et al., 2008). However, the role of group II mGlu receptors in withdrawal appears complex and most likely depends on changes in multiple brain areas.

Although the sequence of events linking mGluR2 adaptations to nAChR activation is unknown, it seems that the reasons for its up- and down-regulation pattern after adolescent nicotine exposure may lie in its function. Metabotropic GluR2 receptors are located on presynaptic glutamatergic terminals where they are activated by glutamate spill over to inhibit glutamate release (Mateo and Porter, 2007). It was shown that activation of mGluR2s can also regulate release of other neurotransmitters: it can inhibit GABA release via a presynaptic mechanism (Bradley et al., 2000; Pilc et al., 2008). Given the inhibitory role of mGluR2 in neurotransmitter release, its function seems to counteract that of nAChR, which enhances both excitatory and inhibitory synaptic transmission (Lambe et al., 2003; Couey et al., 2007; Poorthuis et al., 2012). The short-term effects of adolescent nicotine exposure most likely involve enhanced levels of inhibition in prefrontal network. Accordingly, we found an initial and transient upregulation of inhibitory mGluR2 receptor directly following nicotine exposure during adolescence (Counotte et al., 2011), which would contribute to the same effect.

In general, factors that lead to enhanced excitation can cause alterations in mGluR2 transmission and cause cognitive deficits (Melendez et al., 2004; Pozzi et al., 2011). Enhanced glutamate release in PFC was found to be associated with attention deficit and loss of impulse control (Pozzi et al., 2011). MGluR2 agonists are effective in improving cognitive deficits if enhanced glutamate release is caused by NMDA receptor antagonists (Pozzi et al., 2011). Furthermore, the important role of prefrontal mGluR2 signaling in cognition is stressed by its link to brain disorders such as depression and schizophrenia. Activation of this receptor has even been proposed as a novel treatment approach for these disorders (Gupta et al., 2005; Palucha and Pilc, 2005; Pilc et al., 2008; Conn et al., 2009). Thus, mGluR2 signalling seems to be a good candidate for shaping cognitive behavior and its impairment leads to disturbances in cognitive function.

At the level of synapse function, alterations in mGluR2 levels affect both short-term synaptic plasticity as well as STDP in later life. Short-term depression (STD) is reduced in adult animals as a result of nicotine exposure during adolescence (Counotte et al., 2011). In control animals, blocking mGluR2 signalling with mGluR2 antagonists also results in reduced STD. Reduced mGluR2 signalling after nicotine exposure has a similar effect on STD as mGluR2 block by antagonist (Figure 1). Thereby, mGluR2 may act as an inhibitory feedback mechanism in conditions of excessive excitation and high glutamate release, as occurs when a neuron fires a train of action potentials. Especially at high frequency stimulation the effect of mGluR2 on STD was most prominent at excitatory synapses on layer V pyramidal neurons in the PFC (Counotte et al., 2011). The lasting reduction of mGluR2 levels and function after adolescent nicotine exposure leads to reduced inhibitory feedback on pyramidal cells and reduces the regulatory role of this receptor in short-term plasticity. Most likely, activation of mGluR2s affects presynaptic calcium channel function as was found in the calyx of Held, by direct electrophysiological recordings from presynaptic terminals (Takahashi et al., 1996). Agonists of metabotropic glutamate receptors (mGluRs) suppressed high voltage-activated P/Q-type calcium channels in the presynaptic terminal, thereby inhibiting transmitter release (Takahashi et al., 1996). Since presynaptic Ca^2+^ dynamics play a key role in short—term plasticity (Zucker and Regehr, 2002), decrease in Ca^2+^ current may explain mGluR-dependent modulation of STD.

STD may equip the synapse with low-pass filtering properties, by which the synapse will pass on the first of stimulus in a train of stimuli unaltered, while the rest are attenuated. In this manner it shapes the information transfer by synaptic networks and gives rise to sensory and behavioral phenomena (Zucker, 1989). For example, in somatosensory cortex of rat, *in vivo* whole-cell recordings in cortical neurons during whisker deflection directly demonstrated that synaptic depression of thalamic input to the cortex contributes to rapid adaptation of sensory responses (Chung et al., 2002). Selective attention, the ability of an organism to filter out relevant information in the face of distractors, can build upon just such synaptic process. Layer V pyramidal neurons in PFC handle diverse incoming information from mediodorsal thalamus and from local neurons and these connections are important in mediating executive functions such as for example working memory (Floresco et al., 1999). STD on this level may represent a higher level of sensory adaptation that can be expressed as decreased levels of attention and responsiveness. Reduced short-term plasticity after nicotine exposure compromises the ability of prefrontal neurons to efficiently filter out irrelevant information.

We recently found that glutamatergic synapses in the PFC show increased spike-timing-dependent LTP 5 weeks after nicotine exposure during adolescence (Figure 1) (Goriounova and Mansvelder, 2012). This was not the case when animals were exposed to nicotine during adulthood, indicating that adolescence is a vulnerable period for these lasting changes to occur. The long-term effects on LTP 5 weeks after nicotine exposure during adolescence were opposite to the effects immediately following nicotine exposure during adolescence, where spike-timing-dependent LTP is supressed. Thus, nicotine exposure during adolescence has lasting effects on the mechanisms of STDP and persistent synaptic alterations that increase LTP.

What is the mechanism underlying the long-term effects of nicotine exposure during adolescence on STDP? We hypothesized that altered levels of mGluR2 receptors can explain the lasting effects on STDP. Reduced mGluR2 signalling in adult rats after nicotine exposure (Counotte et al., 2011) may contribute to the decreased plasticity we observed. Blocking mGluR2 receptors with MPPG resulted in increased LTP comparable to levels observed in adult rats treated with nicotine during adolescence (Goriounova and Mansvelder, 2012). In nicotine-treated rats, where the synaptic mGluR2 receptor levels are reduced (Counotte et al., 2011), enhancing mGluR2 activity by applying mGluR2 agonist LY379268 completely abolished LTP (Goriounova and Mansvelder, 2012). Thereby, mGluR2 signalling bidirectionally influences spike-timing-dependent LTP: reducing mGluR2-dependent inhibition leads to increased LTP, while enhancing mGluR2 activation blocked LTP. Thus, immediately following adolescent nicotine exposure, increased levels of mGluR2s may be responsible for reduced LTP induction, and 5 weeks following adolescent nicotine exposure, the lasting reduction in mGluR2 signalling can explain the increased LTP in the adult mPFC.

STDP depends on the precise timing of the synaptic input and the postsynaptic action potential and this temporal relationship resembles typical features of associative learning (Letzkus et al., 2007). Although STDP has not been directly linked to attention performance, the ability to associate goal-relevant information is crucial for any cognitive behavior. In nicotine-treated rats the same amount of pre- and postsynaptic activity leads to more synaptic potentiation. This may suggest that the PFC network would even associate irrelevant stimuli.

## Conclusion

The prefrontal cortex, the brain area responsible for executive functions and attention performance, is one of the last brain areas to mature and is still in the process of developing during adolescence. This places the adolescent brain in a vulnerable state of imbalance, susceptible to the influence of psychoactive substances such as nicotine. In prefrontal networks nicotine modulates information processing on multiple levels by activating and desensitizing nicotine receptors on different cell types and in this way affects cognition. The adolescent brain is particularly sensitive to the effects of nicotine. Studies in human subjects indicate that smoking during adolescence increases the risk of developing psychiatric disorders and cognitive impairment in later life. In addition, adolescent smokers suffer from attention deficits, which progress with the years of smoking.

From studies in the rodent brain it is becoming clear that on the short-term, adolescent, but not adult, nicotine exposure increases the expression of nAChRs containing α4 and β2 subunits in the mPFC, which leads to an increase in nicotine-induced GABAergic synaptic transmission. In addition, mGluR2 levels on presynaptic glutamatergic terminals in the PFC are increased, causing a reduction in glutamatergic synapse strength and reducing STDP (Figure 1). Changes in nAChR levels are reversible: in the adult rodent brain, weeks after nicotine levels have subsided, nAChR levels in the PFC return to baseline levels. In contrast, at this stage, mGluR2 levels have reduced significantly below baseline levels, thereby altering mGluR2 signaling during short-term plasticity, augmenting spike-timing-dependent potentiation and hampering attention performance. This reduction in mGluR2 signaling underlies the reduced attention performance observed in animals after nicotine exposure during adolescence (Counotte et al., 2011). Thereby, mGluR2 signaling could be a therapeutic target for alleviating attention and impulse control problems in later life.

New questions and opportunities arise from these recent findings. The long-term adaptations involving mGluR2s can have profound implications for network functioning and affect more complex levels of information processing. What are the steps that lead from nicotine exposure and nAChR activation in the adolescent brain to adaptations in synaptic mGluR2 levels? Unveiling the signaling routes involved may provide a broader view on the adaptation strategies used during brain development in response to environmental factors. Another interesting question would be whether mGluR2 signaling is involved in a broader spectrum of attention impairments with different etiology. If changes in mGluR2 signaling are a common underlying mechanism for attention malfunction it would make it a suitable pharmacological target for therapy.

### Conflict of interest statement

The authors declare that the research was conducted in the absence of any commercial or financial relationships that could be construed as a potential conflict of interest.
